# Multifocal Congenital Simple Hamartoma of the Retinal Pigment Epithelium: A Multimodal Imaging Case Study

**DOI:** 10.7759/cureus.60755

**Published:** 2024-05-21

**Authors:** Joobin Khadamy, Navid Elmi Sadr

**Affiliations:** 1 Ophthalmology, Skellefteå Eye Clinic, Skellefteå, SWE; 2 Ophthalmology, University Hospital of Umeå, Umeå, SWE; 3 Ophthalmology, Semnan University of Medical Sciences, Semnan, IRN; 4 Clinical Research Development Unit, Kowsar Educational, Research and Therapeutic Hospital, Semnan University of Medical Sciences, Semnan, IRN

**Keywords:** choroidal malignant melanoma, chrpe (congenital hypertrophy of the retinal pigment epithelium), oct (optical coherence tomography), retinal pigment lesion, indocyanine green angiography, fluorescein angiography, optical coherence tomography, multimodal imaging, cshrpe, congenital simple hamartoma of the retinal pigment epithelium

## Abstract

Congenital simple hamartoma of the retinal pigment epithelium (CSHRPE) is a rare benign tumor often detected incidentally during routine eye exams. We present a case of multifocal CSHRPE in a 32-year-old Hispanic woman, emphasizing the diagnostic challenges posed by its presentation and the pivotal role of multimodal imaging in accurate diagnosis. Despite initial difficulties due to a history of trauma and pigmented fundus, advanced imaging techniques, including optical coherence tomography (OCT), OCT angiography (OCTA), fluorescein angiography (FA), and indocyanine green angiography (ICGA), facilitated a precise diagnosis. Notably, OCTA revealed high signal intensity and flow at the largest nodule site while FA and ICGA exhibited characteristic blockage patterns. Moreover, smaller nodules exhibited OCT findings supporting the theory of islands of retinal pigment epithelium (RPE) cells proliferating ectopically within the retina. Our case underscores the importance of comprehensive imaging assessment in distinguishing CSHRPE from other lesions, contributing to a deeper understanding of this rare ocular condition.

## Introduction

Pigmented tumors localized in the posterior segment of the eye exhibit a diverse array of origins and characteristics. These tumors may arise from either the retinal pigment epithelium (RPE) or the choroidal melanocytes and can be categorized as benign or malignant. Among the benign tumors encountered is congenital simple hamartoma of the retinal pigment epithelium (CSHRPE), a rare solitary nodular hyperpigmented lesion typically found at or near the fovea. CSHRPE is frequently detected incidentally during routine eye exams because it typically lacks symptoms and does not exhibit progressive behavior. However, despite its benign nature, diagnosing CSHRPE can still be challenging [1-3].

CSHRPE is believed to have a congenital origin, with its pathogenesis proposed to involve disruptions in the signaling pathways during the migration of RPE cells in embryonic development. It is essential to differentiate between embryogenic RPE migration and secondary RPE migration, which can be triggered by factors such as toxicity, trauma, infectious disease, degenerative or dystrophic diseases, vascular conditions, or pigment clumps observed in diseases like macular telangiectasia (MacTel) [2,3].

While it is reasonable to monitor asymptomatic benign lesions like CSHRPE, it is imperative to swiftly differentiate them from potentially malignant tumors that may mimic their appearance. Here, we present a unique case of multifocal CSHRPE in a Hispanic female, emphasizing the diagnostic value of various imaging modalities such as red-free (RF), infrared (IR), enhanced IR, shadowgram, optical coherence tomography (OCT), OCT angiography (OCTA), fluorescein angiography (FA), and indocyanine green angiography (ICGA). This case underscores the significance of comprehensive imaging assessment in achieving precise diagnoses. It sheds light on the diverse clinical presentations of CSHRPE, further enriching our understanding of this rare ocular condition.

## Case presentation

**Figure 1:**
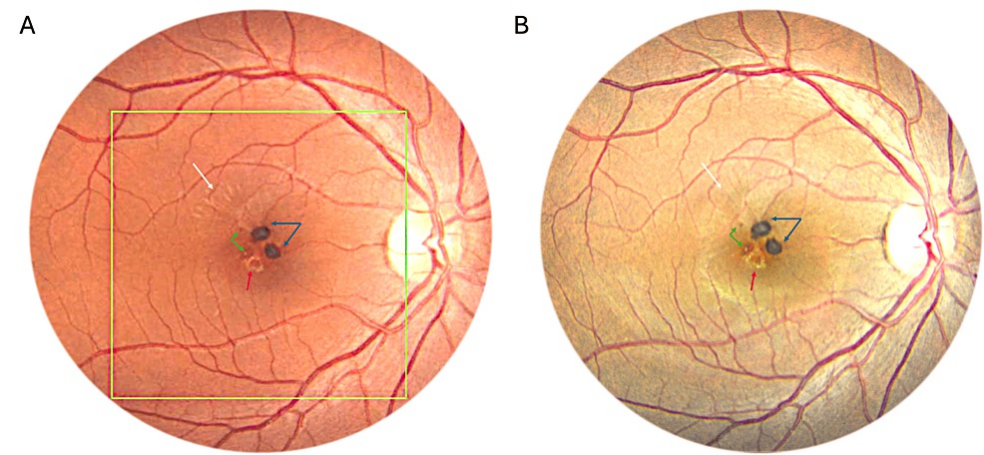
Fundus photographs of the right eye showing stable findings two months later Initial examination of the right eye (A) and follow-up after two months (B) reveal consistent findings, including multiple pigmented nodules (two prominently highly pigmented (blue arrows) and two smaller, less pigmented nodules (green arrows)), a yellow ring encircling a pigmented area inferior to the foveal center (red arrow), and radial retinal wrinkles (white arrows).

A 32-year-old woman presented to the eye clinic with a complaint of two days of visual disturbance in her right eye. She had no significant systemic or ocular history but reported experiencing minor blunt contusion trauma to the same eye from her baby, who accidentally struck her eye. Despite this, she denied pain and any other associated symptoms. On examination, her visual acuity was 20/20 in both eyes, and intraocular pressure and anterior segment were normal. Fundus examination of the right eye revealed multiple hyperpigmented elevations in the parafoveal region, and yellow hyperreflective materials near the macula center (Figure 1A), which remained unchanged after two months (Figure 1B).

**Figure 2:**
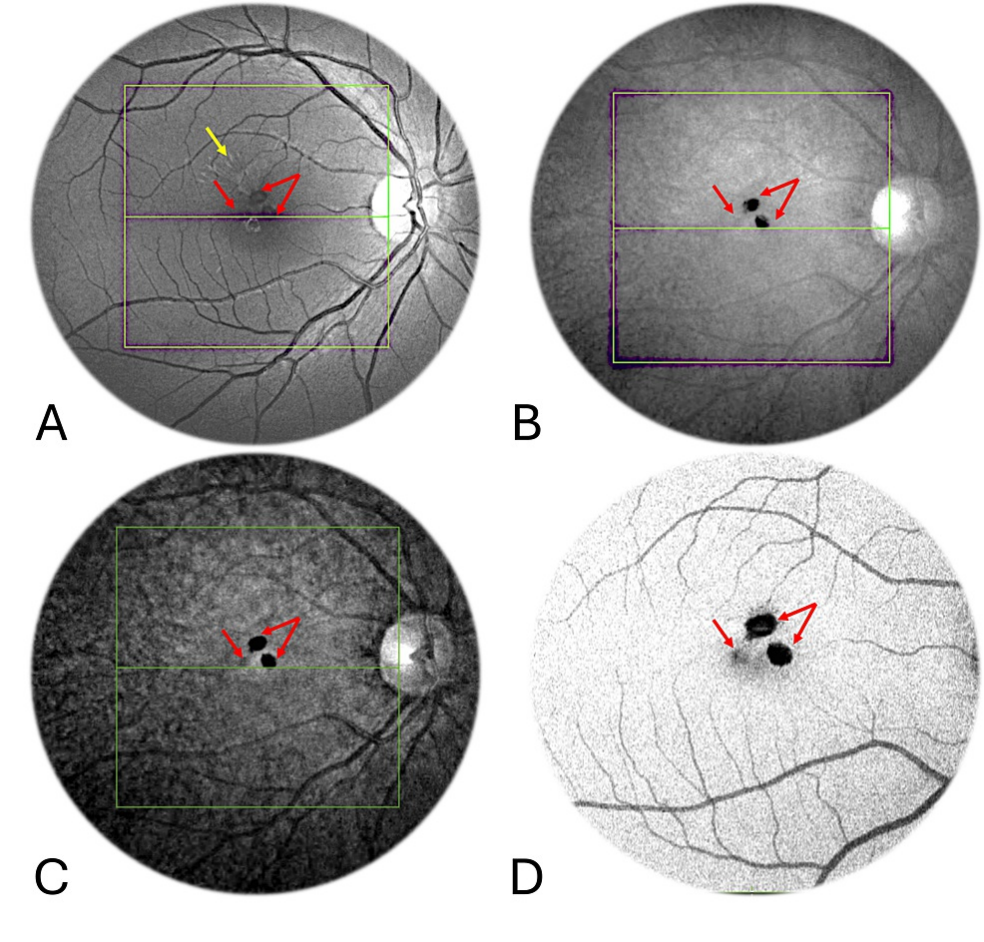
Multimodal imaging of lesions including red-free, infrared, enhanced infrared, and shadowgram The hyperpigmented lesions appeared dark (red arrows) in the red-free (A), infrared (B), enhanced infrared (C), and shadowgram (D) images. The red-free image accentuated the radial folds on the internal surface of the retina (yellow arrow in A).

These hyperpigmented lesions appeared dark in various imaging modalities, including red-free (RF) (Figure 2A), infra-red (IR) (Figure 2B), enhanced IR (Figure 2C), and shadowgram (Figure 2D).

**Figure 3:**
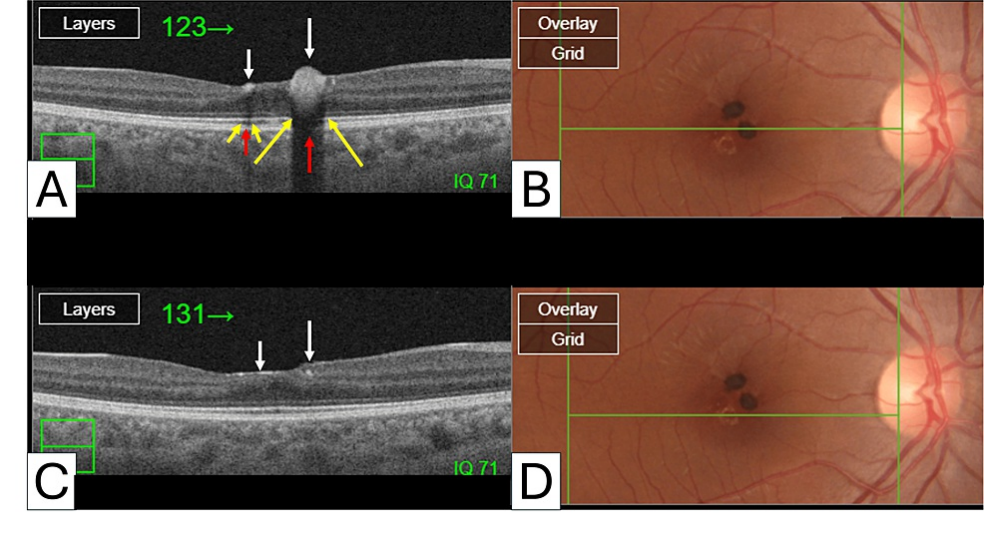
Optical coherence tomography of congenital simple hamartoma of the retinal pigment epithelium Optical coherence tomography scans (A and C) depicting hyperpigmented nodules characteristic of congenital simple hamartoma of the retinal pigment epithelium. The horizontal green line in the color photos (B and D) demarcates the B-scan intersection line of the pigmented lesions. Notably, at the lesion site, hyperreflective lesions protrude toward the vitreous cavity (white arrows), accompanied by posterior shadowing that induces abrupt hyporeflectivity (red arrows). This phenomenon manifests as a pseudo-disruption of the photoreceptor and retinal pigment epithelium layer (yellow arrows).

OCT imaging revealed the protrusion of hyperreflective lesions toward the vitreous cavity, accompanied by posterior shadowing that resulted in abrupt hyporeflectivity, giving the appearance of pseudo-disruption of the photoreceptor and retinal pigment epithelium (RPE) layer (Figure 3).

**Figure 4:**
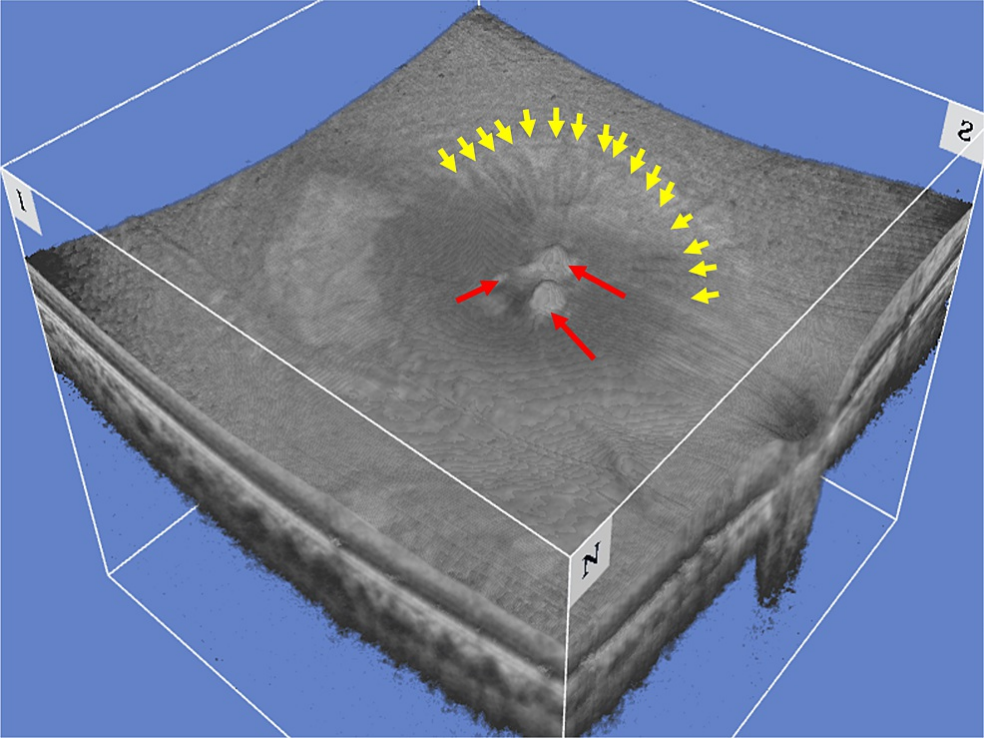
Three-dimensional rendered optical coherence tomography Three-dimensional rendered optical coherence tomography showcased the protrusion of pigmented nodules toward the vitreous (red arrows), along with radial wrinkles in the internal limiting membrane (yellow arrows).

Three-dimensional (3D) rendered OCT enhanced the protrusion of pigmented nodules toward the vitreous, along with radial wrinkles in the internal surface of the retina (Figure 4).

Despite initial suspicion of a preretinal hemorrhage, the stability of the lesions prompted further evaluation with OCTA, FA, and ICGA.

**Figure 5:**
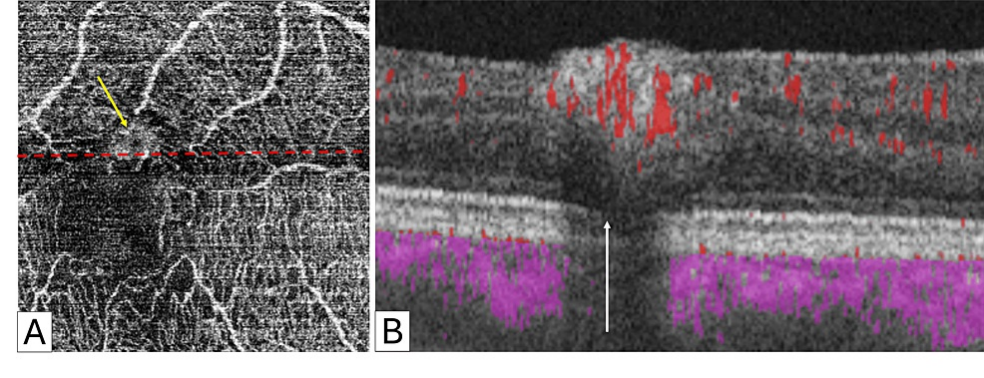
Optical coherence tomography angiography of congenital simple hamartoma of the retinal pigment epithelium In the superficial capillary plexus (SCP) slab of optical coherence tomography angiography (OCTA), a high signal intensity (yellow arrow) is observed at the site of the largest pigmented nodule (A). Additionally, the intersection of the lower part of the same nodule (red dashed line in A) exhibits flow (red dots) in the flow Angio B image (B). The posterior shadowing of the nodule reduces the retinal pigment epithelium and receptor layers' signals (white arrow). In Angio B images, blood flow signals are superimposed on the B-scan, with flow signals above the RPE shown in red and those below displayed in pink.

OCTA was inconclusive and showed high signal intensity and high flow at the site of the largest nodule (Figure 5).

**Figure 6:**
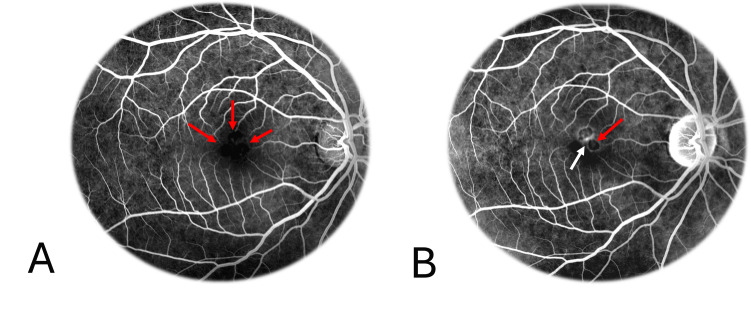
Fluorescein angiography in congenital simple hamartoma of the retinal pigment epithelium Fluorescein angiography exhibited blockage at the site of the hyperpigmented lesions (red arrows), with no evidence of early leakage (A) but late ring-form hyperfluorescence staining (B) between the nodules (white arrow).

FA exhibited blockage at the site of the hyperpigmented lesions, with no evidence of leakage but late ring-form hyperfluorescence staining between the nodules (Figure 6).

**Figure 7:**
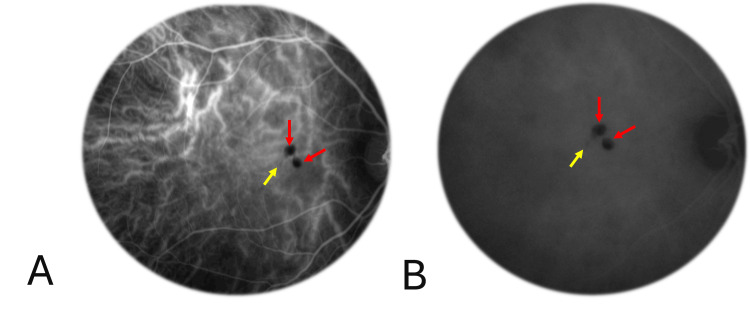
Indocyanine green angiography in congenital simple hamartoma of the retinal pigment epithelium Early indocyanine green angiography (A) demonstrated hypocyanescence due to blockage at the site of the hyperpigmented lesions (red arrows), with no evidence of leakage or staining in the late phase (B). Although smaller pigmented nodules were hardly distinguishable on early ICGA images (A), one of them (yellow arrows) showed a more distinct blockage in the late phase (B).

ICGA demonstrated blockage at the site of the hyperpigmented lesions, with no evidence of leakage (Figure 7).

The characteristic findings on multimodal imaging led to the diagnosis of CSHRPE, and the patient was scheduled for further follow-up. Written consent was obtained from the patient, and the report adheres to the principles outlined in the Helsinki Protocol.

## Discussion

CSHRPE manifests as a discernible nodule that protrudes through the sensory retina, sometimes extending into the vitreous cavity. Typically, this lesion impacts the entire thickness of the retina and is frequently situated in the central macular area, notably near the fovea and adjacent to the foveola [2-5].

Despite its proximity to the foveola, patients with CSHRPE often maintain normal visual acuity. CSHRPE typically maintains a stable size and demonstrates a benign course, suggesting a watchful waiting approach for long-term follow-up. However, compromised vision has been reported in cases where the tumor is located near the fovea [6], leading to extensive foveal traction [2], or complications related to the tumor itself. Additionally, associations with macular edema, vitreomacular traction, epiretinal membrane, and a macular hole have been documented in cases of CSHRPE [7-11]. In such cases, interventions such as anti-vascular endothelial growth factor (anti-VEGF) injections or vitrectomy have been performed with promising effects [7,10].

Furthermore, CSHRPE has been reported as a coincidental finding alongside Coat’s disease or the presence of a congenital retinal arterial macrovessel crossing the macular region in the same eye [12-13]. Despite these associations, the precise relationship between these conditions remains unclear. No systemic associations with systemic tumors are reported in contrast to atypical congenital hypertrophy of the retinal pigment epithelium (CHRPE).

Shields et al. reported associated features in a series of five cases of CSHRPE [2], including minimally dilated retinal feeding arteries and draining retinal veins (100%), mild adjacent retinal traction (80%), retinal exudation (20%), and vitreous pigmented cells (20%). Additionally, Badawi et al. documented a case of CSHRPE with a solitary tumor associated with multiple posterior pole small hyperpigmented lesions and peripheral hypopigmented lesions indicating RPE atrophy [14].

The differential diagnosis of CSHRPE encompasses several conditions, including CHRPE, combined hamartoma of the retina and RPE, adenoma or adenocarcinoma of the RPE, retinal invasion from underlying choroidal nevus or melanoma, and intraretinal foreign body [2,5,15,16]. Choroidal nevi typically have less well-defined borders compared to CSHRPE, whereas combined hamartoma of the retina and RPE display a grayish coloration and tortuous intraretinal vasculature. Adenoma or adenocarcinoma of the RPE may bear resemblance to CSHRPE but often manifests with prominent feeding and draining blood vessels, as well as intra- and sub-retinal exudation. Unlike the stable nature of CSHRPE, retinal invasion from underlying choroidal nevus or melanoma tends to exhibit growth features and changes over time [2,5,15,16].

The rarity of CSHRPE, along with its asymptomatic nature, particularly in patients with a history of trauma, pigmentary variations [4,14], or exposure to Toxoplasma gondii parasite or other infectious diseases complicates its accurate diagnosis during the initial evaluation. However, advanced imaging modalities play a crucial role in differentiation, offering valuable insights into the structural aspects of CSHRPE.

Initially, the case was suspected to involve preretinal hemorrhage from trauma, leading to delayed accurate diagnosis; however, follow-up imaging confirmed the diagnosis. Our imaging findings are consistent with previous reports, although there is limited literature detailing the appearance of the lesion in IR and near-IR, and few reports describe both hyper- and hyporeflective characteristics [9]. In prior studies, the tumor often appeared reddish in multicolor fundus photos [9], a feature we did not evaluate in our study. Modifying the contrast and brightness of the captured images could potentially enhance the visibility of the pigmented nodules. Additionally, our investigation revealed that 3D-rendered OCT imaging provided improved visualization of radial wrinkles on the internal retinal surface, which were not easily discernible in B-scan OCT images.

FA may display hypofluorescence in the early stages and a halo of fluorescence in the later stages. ICGA findings are often lacking in reports, with hypocyanescence indicating blockage observed in this case. FA may also reveal feeding and draining vessels of the tumor [9]. OCTA imaging may uncover tangled or radial intrinsic microvasculature within the tumor, a feature not observable using FA and ICGA imaging techniques [4,17]. Moreover, the tumor appeared hypo-autofluorescent in fundus autofluorescence (FAF) imaging, likely due to melanin-rich CSHRPE blocking lipofuscin-originated autofluorescence at the tumor site [4,18]. CSHRPE is typically too small to be visualized using ultrasonography. However, in specific instances, B-scan ultrasonography may reveal an echogenic, nodular pattern without orbital shadowing [2,11].

OCT, in conjunction with FA, is pivotal in imaging assessment for CSHRPE. Typically, OCT reveals a hyperreflective nodule with posterior shadowing. Both CSHRPE and bone spicule pigments (BSP) in retinitis pigmentosa (RP) stem from the intraretinal proliferation of migrated RPE cells. However, while CSHRPE is congenital, BSP is a secondary effect of RP. If RPE cell proliferation occurs around a major retinal vessel branch, resembling BSP in RP, it may manifest as a hyperreflective cuff or donut encircling the vessels in B-scan OCT, with or without central hyporeflectivity at the vessel lumen [19].

Multifocal electroretinography (mfERG) and microperimetry findings in CSHRPE do not consistently demonstrate characteristic features of the tumor, primarily due to the lesion being smaller in size than the stimulating spot size. While some studies report normal mfERG patterns despite reduced vision [6], others note a marked reduction in central amplitude [14]. Microperimetry and adaptive optics have been used to assess retinal sensitivity and photoreceptor arrangement in CSHRPE cases, revealing consistent sensitivity responses over the pigmented tumor and focal decreases in retinal sensitivity in areas of RPE atrophy adjacent to the tumor [4].

The histopathologic characteristics are not fully investigated. However, a single report of histopathologic examination of an excised lesion revealed hyperplastic RPE cells with fibrous metaplasia, accompanied by speculated hyalinized vascular components. Notably, some lesions may be located in the pre-retinal region and may not fully penetrate the retinal thickness, differing from prior findings [10].

In contrast to previous reports where CSHRPE presented as a solitary mass, our case revealed two separate heavily pigmented masses and smaller, less pigmented intraretinal masses in close proximity to each other [20]. The presence of less pigmented areas with hyperreflectivity and posterior shadowing in our case may suggest secondary structural changes surrounding the tumor or the presence of another cluster of tumor cells. If these areas are considered a nidus of the tumor, it could imply that the tumor is not full-thickness but comprises islands of RPE cells that have proliferated ectopically within the retina [10]. However, without access to pathological samples, further histopathological investigation is needed to clarify this aspect.

## Conclusions

In conclusion, the history of trauma and the presence of multifocal nodules delayed the diagnosis since it challenged the conventional understanding of CSHRPE as a solitary mass. This case report underscores the crucial role of advanced imaging modalities like OCT and FA in accurately diagnosing CSHRPE. Furthermore, the OCT findings in smaller pigmented nodules support the theory of islands of RPE cells proliferating ectopically within the retina instead of a full-thickness tumor. These insights prompt further histopathological or adaptive optic studies to validate these findings and elucidate the underlying mechanisms of this rare ocular condition.
